# The Zinc Sensing Receptor, ZnR/GPR39, in Health and Disease

**DOI:** 10.3390/ijms19020439

**Published:** 2018-02-01

**Authors:** Michal Hershfinkel

**Affiliations:** Department of Physiology and Cell Biology and The Zlotowski Center for Neuroscience, Faculty of Health Sciences, POB 653, Ben-Gurion Ave. Ben-Gurion University of the Negev, Beer Sheva 84105, Israel; hmichal@bgu.ac.il, Tel.: +972-8-6477318

**Keywords:** zinc, ZnR/GPR39, zinc signaling, neuron, keratinocyte, epithelium, intestine, colon, bone

## Abstract

A distinct G-protein coupled receptor that senses changes in extracellular Zn^2+^, ZnR/GPR39, was found in cells from tissues in which Zn^2+^ plays a physiological role. Most prominently, ZnR/GPR39 activity was described in prostate cancer, skin keratinocytes, and colon epithelial cells, where zinc is essential for cell growth, wound closure, and barrier formation. ZnR/GPR39 activity was also described in neurons that are postsynaptic to vesicular Zn^2+^ release. Activation of ZnR/GPR39 triggers Gαq-dependent signaling and subsequent cellular pathways associated with cell growth and survival. Furthermore, ZnR/GPR39 was shown to regulate the activity of ion transport mechanisms that are essential for the physiological function of epithelial and neuronal cells. Thus, ZnR/GPR39 provides a unique target for therapeutically modifying the actions of zinc in a specific and selective manner.

## 1. Introduction

The symptoms of zinc deficiency are particularly prominent in the digestive, immune, nervous, endocrine, and integumentary systems [1,2,3,4,5]. In many cases dietary zinc supplementation can ameliorate the symptoms and indeed zinc supplementation is widely used to treat diarrhea, the common cold, and skin conditions. The mechanisms underlying the roles of zinc have been revealed in the last two decades, but there is still a lot to learn about the pathways and regulation of zinc ions (Zn^2+^). Initially, Zn^2+^ was identified as a structural element and cofactor in enzymes [6,7] and transcription factors [8,9,10]. It is estimated that about 3000 proteins contain Zn^2+^ binding sites, and interaction with Zn^2+^ regulates or modulates the activity of these proteins, thereby affecting numerous cellular processes [11]. Cellular Zn^2+^ is associated with these proteins with a very high affinity and is considered a tightly bound pool of Zn^2+^ [10,12]. The labile Zn^2+^ pool in cells includes proteins that interact with Zn^2+^ via histidines, cysteines, or glutamate/aspartate residues; most prominent are the metallothioneiens (MTs) Zn^2+^ binding proteins [13]. This is a dynamic pool that releases Zn^2+^ upon redox signaling and oxidative or nitrosative stress, and contributes to cellular signaling [14,15,16,17,18]. In addition, cytosolic Zn^2+^ rise, likely mediated by Zn^2+^ transporters on the endoplasmic reticulum (ER), was monitored in mast cells following activation of the immunoglobulin receptor [19,20]. Subsequent studies determined that Zn^2+^ transporters found on various cellular organelles induce changes in cytosolic or organellar Zn^2+^ and thereby modulate cellular signaling [21,22,23,24,25,26]. Indeed, Zn^2+^ transport from the ER, Golgi, or mitochondria plays an important role in the function of mammary gland or prostate epithelial cells and other secretory cells [27,28,29]. Similar release of Zn^2+^, from the ER, during cardiac function regulates Ca^2+^ leakage from the ER in these cells [30,31]. These studies established Zn^2+^ as a second messenger that is released following diverse stimuli and triggers the regulation of kinases or phosphatases as well as protein expression [20,32]. Cellular Zn^2+^ is buffered by interaction with proteins and formation of complexes to rapidly reduce levels of Zn^2+^ to the picomolar range [17,33]. Importantly, transient changes in extracellular levels of Zn^2+^ can also occur following release of Zn^2+^-containing vesicles. Such vesicular Zn^2+^ is found in neurons, epithelial Paneth cells of the intestine or the salivary gland, as well as in pancreatic β-cells [34]. The vesicular Zn^2+^ can be released during normal activity of the cells; for example, Zn^2+^ is released into the synapse during neuronal activity or is secreted from β-cells or mammary epithelial cells [35,36,37,38,39,40]. Release of Zn^2+^ from cells can also occur following cellular injury and cell death, which liberates Zn^2+^ from the numerous Zn^2+^-binding proteins or cellular organelles [41]. Extracellular Zn^2+^ can interact with specific binding sites on numerous proteins and regulate their activity. For example, extracellular Zn^2+^ allosterically modulates numerous neuronal receptors, i.e., *N*-methyl-d-aspartate (NMDA), γ-Aminobutyric acid (GABA), or glycine receptors, thereby modulating the excitatory and inhibitory responses [42,43,44,45,46]. In epithelial cells, extracellular Zn^2+^ regulates the activity of purinergic receptors and the store-operated Ca^2+^ (SOC), representing an important link between Zn^2+^ and intracellular Ca^2+^ [47,48,49]. Application of Zn^2+^ was also suggested to upregulate the phosphatidylinositol-4,5-bisphosphate 3 (PI3) kinase/AKT pathway [50] or mitogen-activated protein kinases (MAPKs) [51], both essential to cell survival and proliferation.

## 2. Identification of a Zn^2+^-Sensing Receptor, ZnR/GPR39

In addition to the large numbers of Zn^2+^ homeostatic proteins described above, a distinct target for extracellular Zn^2+^ is the plasma membrane G-protein coupled receptor that is sensitive to Zn^2+^, ZnR/GPR39 [52,53,54]. G-protein coupled receptors are a large family of seven-transmembrane proteins that mediate cellular signaling in response to a diverse array of extracellular stimuli [55]. The endogenous Zn^2+^, released during physiological activity, acts as a first messenger and triggers intracellular Ca^2+^ signaling via the specific Gαq-coupled receptor ZnR/GPR39 [34,56]. Activity of ZnR/GPR39 in tissues relevant to Zn^2+^ signaling has been identified in neurons, colon epithelial cells (colonocytes), skin epidermal cells (keratinocytes), pancreatic cells, prostate cancer cells, salivary gland cells, and in bones [57,58,59,60,61]. In neurons, stimulation of the mossy fibers triggers ZnR/GPR39-dependent Ca^2+^ rises in postsynaptic CA3 (Cornu Ammonis 3) neurons [62] that are diminished in the presence of a non-permeable Zn^2+^ chelator, or in the absence of the Zn^2+^ transporter-3 (ZnT3), which is responsible for synaptic Zn^2+^ accumulation. Similar ZnR/GPR39 responses were observed in postsynaptic neurons of the auditory brainstem nucleus, the dorsal cochlear nucleus [63]. Importantly, ZnR/GPR39 activity was shown to enhance neuronal inhibitory tone, and zinc deficiency is associated with epilepsy and seizures, suggesting the significant physiological role of ZnR/GPR39 [53,64,65,66,67,68]. Luminal application of Zn^2+^ to colon epithelial cells, colonocytes, was sufficient to activate the plasma membrane ZnR/GPR39 [69], which is highly expressed in this tissue [70,71]. In colonocytes, ZnR/GPR39 activated cellular pathways that are strongly associated with cell growth, MAP, and PI3 kinases. The prominent role of zinc supplementation in digestive system function, taste disorders, and salivary secretion suggests that ZnR/GPR39 may play an important role in the physiological functions of this system. A specific role for zinc in wound healing and the strong link between its deficiency and skin lesions suggested that ZnR/GPR39 may mediate cell proliferation and wound healing, thereby contributing to skin health. A recent study also describes ZnR/GPR39 expression in the oviduct, where it colocalized with a higher concentration of Zn^2+^ but its activity has not been studied [72]. While a link to Zn^2+^ physiology is still not clear, ZnR/GPR39 was also associated with adipocyte and myoblast proliferation and differentiation [73,74]. Activation of ZnR/GPR39 was triggered by transient changes in extracellular Zn^2+^. While exogenous application of Zn^2+^ may trigger ZnR/GPR39 activation, the endogenous sources of vesicular Zn^2+^ may be the physiological trigger of ZnR/GPR39 activation, i.e., Zn^2+^ released from neuronal vesicles, salivary gland vesicles, pancreatic enzymes, or Paneth cells in the intestinal epithelium [35,36,37,38,39,40,75]. In addition, extracellular Zn^2+^ levels may transiently change following efflux mediated by Zn^2+^ transporters, such as ZnT6 [76], or following injury and cell death [41].

## 3. ZnR/GPR39-Dependent Signaling

**Figure 1:**
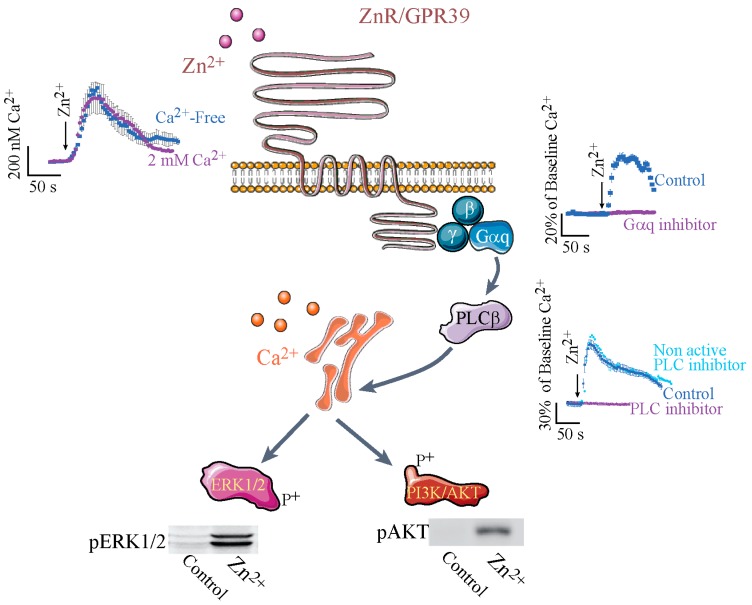
Schematic representations of common Zn^2+^ sensing receptor, ZnR/GPR39, signaling in epithelial cells. Extracellular signal–regulated kinases, ERK; Phosphatidylinositol-4,5-bisphosphate 3 (PI3) kinase/AKT, PI3K/AKT; Phospholipase C, PLC.

Intracellular Ca^2+^ signaling triggered by extracellular Zn^2+^ was the first functional identification of a distinct Zn^2+^ sensing receptor, named ZnR [77]. Use of pharmacological inhibitors of Gαq [78,79], inositol 1,4,5-trisphosphate (IP3) receptor and the phospholipase C (PLC), indicated that a Zn^2+^-dependent Ca^2+^ rise is mediated by activation of a Gαq-coupled receptor, such that the Ca^2+^ is released from thapsigargin-sensitive stores following activation of the IP_3_ receptor [52,57] (see Figure 1). Importantly, the Zn^2+^-dependent signaling was mediated by changes in extracellular, and not intracellular, levels of this ion, as expected from a G-coupled receptor [52,57]. The search for the protein that mediates Zn^2+^-dependent signaling focused on members of the Gαq family of receptors, their possible isoforms, or interactions between these receptors that may affect the affinity towards Zn^2+^; the main candidate in this family was the Ca^2+^-sensing receptor (CaSR). Most G-protein coupled receptors are activated by peptides and not cations, but a CaSR was already identified and its physiological significance to cellular signaling was established [80,81]. The similarity of the ligands and the signaling pathway activated by the CaSR and the putative ZnR suggested that these may be isoforms of the same receptor. Surprisingly, Zn^2+^ turned up in a screen of serum for the agonist of GPR39, which was an orphan receptor until then [82], subsequent studies confirmed that ZnR and GPR39 are one receptor, termed ZnR/GPR39. Despite their ligand similarity, CaSR and GPR39 are not members of the same subfamily of G-protein coupled receptors. The GPR39 is a member of ghrelin receptor family A, while CaSR is a member of family C of the G-protein coupled receptors [83]. It is important to note that ZnR/GPR39 is not activated by extracellular Ca^2+^, nor is the CaSR activated by Zn^2+^ [52,84]. Nevertheless, the affinity of ZnR/GPR39 to Zn^2+^ is modulated by Ca^2+^, as the K_0.5_ of ZnR/GPR39 in salivary gland cells was ~55 µM in the presence of Ca^2+^ and only ~36 µM in its absence [58]. This may be mediated by a direct effect of CaSR on ZnR/GPR39 conformation or its membrane expression or by a direct effect of Ca^2+^ on the Zn^2+^-binding site. Indeed, ZnR/GPR39 and the CaSR have been shown to directly interact in an exogenous overexpression system and may thereby modulate cation-dependent signaling in many systems where they are both expressed [84]. Importantly, the orphan receptor GPR39 was initially suggested to mediate signaling triggered by the obesity-related peptide obestatin [85]. These results were not reproduced by other laboratories and a study using serum identified Zn^2+^ as the endogenous ligand of GPR39 [82]. Using silencing and overexpression, it has been shown that the endogenous Zn^2+^-dependent signaling is mediated by GPR39, which is highly selective for Zn^2+^ and is not activated by Mn^2+^, Cu^2+^, or Fe^2+^ [52,53]. The affinity of ZnR/GPR39 to Zn^2+^ was physiologically adapted to the relevant tissues. For example, Zn^2+^ concentration in the digestive system lumen may reach hundreds of µM [86,87,88] and the colonocytic ZnR/GPR39 has an EC50 (half maximal effective concentration) of 80 µM [52,57]. Physiological relevance was further established when Zn^2+^ release from Caco-2 colonocytes was sufficient to induce ZnR/GPR39-dependent cell growth and tight junction formation [69,89]. In addition, in a cholera toxin model of diarrhea or a dextran sodium sulfate model of colitis, ZnR/GPR39-dependent pathways were not activated following dietary Zn^2+^ depletion [90,91]. Similarly, in the prostate, where there are high concentrations of Zn^2+^/citrate complex and transient release of this ion is likely to occur following cell death or changes in pH, ZnR/GPR39 is adapted to the relevant concentrations, which range from 10 to 200 μM [59]. In contrast, in keratinocytes ZnR/GPR39 EC50 to Zn^2+^ is in the nanomolar range, likely because this tissue contains much lower concentrations of labile Zn^2+^ [41]. Most importantly, the ZnR/GPR39 is triggered during keratinocytic injury, as shown using a scratch assay [41]. In addition, the neuronal ZnR/GPR39 has an affinity that is adapted to the release of Zn^2+^ from the synaptic mossy fiber terminals, and indeed very mild activation of these fibers induces sufficient Zn^2+^ levels to trigger postsynaptic ZnR/GPR39 signaling [62,92]. The differences in the affinity of the ZnR/GPR39 may result from its interaction with other, physiologically relevant G-protein coupled receptors in the tissues, as has been established for many receptors of this family [93].

Since Zn^2+^ can interact with numerous intracellular and extracellular proteins, application of this ion to study the effects of ZnR/GPR39 may yield confusing results and distinct agonists or antagonists would be of importance. Using various screening methods, agonists for ZnR/GPR39 have been suggested but very few were successfully tested in endogenous tissues. A recent study identified several compounds that may interact with ZnR/GPR39 and were shown to affect gastric function in wild-type but not *GPR39* knockout mice, yet these compounds only potentiated the response of the ZnR/GPR39 to Zn^2+^ itself [94]. The use of molecular approaches to modulate expression of ZnR/GPR39, together with pharmacological inhibition of its signaling pathway, is therefore still important to study the effects of ZnR/GPR39. Indeed, the first description of the role of ZnR/GPR39 was established using a knockout mouse, which exhibited accelerated gastric emptying and increased body weight and fat composition [70]. This phenotype strengthened the link between the receptor and the well-known effects of Zn^2+^ on the gastrointestinal system. Future studies using knockout mice required challenging the mice to trigger a phenotypic distinction from the wild-type mice, suggesting that ZnR/GPR39 has a role in stress conditions. Finally, overexpression of ZnR/GPR39 in exogenous systems resulted in signaling that exhibited constitutive activity or was suggested to trigger Gαs or Gα12/13 signaling and CRE- or SRE-dependent gene expression [83], but the physiological significance of these pathways is yet to be determined.

Activation of the Gαq is triggering PLCβ activation and subsequent Ca^2+^ release from thapsigargin-sensitive ER stores. Insets show the Fura-2 fluorescent signals in cells expressing ZnR/GPR39 following application of Zn^2+^. The top left inset shows the calibrated level of Ca^2+^ change, monitored with Fura-2, obtained in the presence or absence of extracellular Ca^2+^; the right upper inset shows the % change of Ca^2+^ levels, relative to baseline Fura-2 fluorescence, in the presence or absence of the Gαq inhibitor (YM-254890); and the right bottom panel shows the % change of Ca^2+^ levels in the presence of the PLC inhibitor (U73122 active form, or U73343 inactive form). Subsequent to the Ca^2+^ signal ERK1/2 (extracellular regulated kinase) or AKT phosphorylation is monitored (shown in blots in the lower panels), indicating activation of the MAPK or PI3K pathways, respectively. (The figure was composed using Servier Medical Art templates (http://smart.servier.com/)).

Subsequent to the Ca^2+^ rise, ZnR/GPR39-triggers activation of the ERK/MAPK and AKT/PI3K pathways [57,84] that are essential for cell survival and proliferation [95]. ZnR/GPR39 activation in keratinocytes, colonocytes, and prostate cancer cells was shown to upregulate ERK and AKT phosphorylation and thereby cell growth. Activation of the Zn^2+^-dependent Ca^2+^ response was first shown to activate ERK1/2 phosphorylation, which was attenuated by functional de-sensitization of ZnR/GPR39, critical for protecting cells from excessive activation of the signaling [84]. In androgen-insensitive prostate cancer cell lines, ZnR/GPR39 activation by Zn^2+^ triggers PI3K pathway upregulation, which is reflected by increased expression and phosphorylation of AKT [84], associated with more malignant phenotypes of carcinomas [96,97,98]. Butyrate is a short-chain fatty acid found to affect colon epithelial cell growth and carcinogenesis [99,100,101,102]. In the colonocytic cell line, butyrate-induced apoptosis was attenuated by ZnR/GPR39-dependent activation of MAPK and PI3K pathways that increased expression of the pro-survival protein clusterin [69]. Moreover, enhanced cell proliferation was monitored using BrdU in colon tissue from ZnR/GPR39 expressing mice, but not in *GPR39* knockout mice, during recovery from treatment with the toxin dextran sodium sulfate [90]. Under normal conditions BrdU staining in knockout mice lacking ZnR/GPR39 did not show differences from the wild-type tissue, suggesting that the baseline proliferation is intact, in agreement with the mild phenotype of these mice. The requirement for enhanced proliferation following the injury is the process that is impaired in the absence of ZnR/GPR39. As such, a role for ZnR/GPR39 may also underlie the healing effects of Zn^2+^ on gastric ulcers [103]. Topical application of zinc-containing ointments to enhance wound healing and re-epithelialization of the skin is well established [104,105,106,107]. Indeed ZnR/GPR39 activation in keratinocytes was shown to trigger MAPK phosphorylation and increased rate of scratch closure, suggesting that the receptor may mediate the effects of Zn^2+^ [41]. Finally, pre-adipocyte proliferation and differentiation are also induced following AKT activation, associated with ZnR/GPR39 expression [73,108]. In neurons, ZnR/GPR39 and subsequent Ca^2+^ release are essential for activation of MAPK by Zn^2+^ [92,109]. Such activation of the MAPK pathway by metabotropic signaling mediates changes in synaptic plasticity [110,111]. Finally, activation of ZnR/GPR39 in a salivary gland ductal cell line was shown to induce ATP release that mediated metabotropic signaling via the purinergic system in neighboring smooth muscle cells [58]. Thus ZnR/GPR39 has paracrine effects on neighboring cells, which may provide an important mechanism by which Zn^2+^ can affect physiological processes in tissues where not all cells express ZnR/GPR39 itself.

Zn^2+^, in contrast to most ligands of G-protein coupled receptors, is not rapidly degraded and a desensitization mechanism to protect cells from excessive Ca^2+^ signals is important for the regulation of ZnR/GPR39 signaling. Indeed, profound and prolonged desensitization [112] is monitored following exposure to subtoxic concentrations of Zn^2+^ [57,59,92]. The desensitization of ZnR/GPR39 by prolonged Zn^2+^ treatment induces internalization and possible degradation of the receptor, and profound loss of ZnR/GPR39 signaling is sustained for several hours. As such, Zn^2+^-induced desensitization was also used to specifically identify the roles of Zn^2+^ via ZnR/GPR39. For example, following ZnR/GPR39 desensitization the Zn^2+^-dependent ERK1/2 phosphorylation was diminished in prostate cancer cells [59]. The pathways that lead to ZnR/GPR39 desensitization are not fully understood. Recruitment of β-arrestin following ZnR/GPR39 with an allosteric modulator in the presence of Zn^2+^ did not induce desensitization but inhibition of Rho kinase blocked this process [113]. 

Zn^2+^ binding to ZnR/GPR39 occurs via two histidine residues, His17 and His19 [114], and an aspartate residue, Asp313. The pH sensitivity of these residues matched the regulation of ZnR/GPR39 response by extracellular pH. The ZnR/GPR39-dependent Ca^2+^ response and subsequent phosphorylation of MAP or PI3 kinase is completely abolished at pH 6.5 [41,109,115]. Hence, ZnR/GPR39 activity is regulated by physiologically relevant changes in extracellular Zn^2+^ or pH [115]. Thus, ZnR/GPR39 may be the mediator for many of the well-established, health-promoting functions of Zn^2+^ [116]. In contrast, local pH changes during inflammatory bowel disease may attenuate ZnR/GPR39-dependent cell proliferation in the digestive system and may contribute to epithelial erosion and barrier breakdown [117].

## 4. ZnR/GPR39 Regulation of Physiological Functions

### 4.1. ZnR/GPR39 Regulates Ion Transport Mechanisms

Downstream to activation of ZnR/GPR39, it has been shown that transport of Na^+^, K^+^, and Cl^−^ are regulated. The movement of these ions is essential for the physiological functions of epithelial cells and neurons. 

The ubiquitously expressed Na^+/^H^+^ exchanger (NHE) is upregulated following cytoplasmic acidification, to induce recovery of intracellular pH [118]. Activation of ZnR/GPR39 upregulates NHE activity in colonocytes, keratinocytes, and neurons [41,57,69,89,109], thereby providing a Zn^2+^-dependent homeostatic mechanism. Colonocytes are constantly exposed to cellular acidification, for example by short-chain fatty acid penetration [119], which can be recovered by NHE activity. Indeed, activation of ZnR/GPR39 in colonocytes and native colon tissues induced activation of NHE, downstream to the Ca^2+^ signaling and ERK1/2 activation, which enhanced the recovery of the colonocytic pH [57,69]. Thus, ZnR/GPR39 plays a role in pH homeostasis that is essential for colonocytes’ survival. Importantly, Na^+^-dependent H^+^ export can lower the extracellular pH. In keratinocytes, ZnR/GPR39 upregulation of NHE activity was also mediated via the same signaling pathway [41]. The extracellular acidification triggered by ZnR/GPR39-dependent activation of NHE may be required for migration of cells during wound healing or for the formation of an effective permeability barrier [120,121]. Intracellular acid loading in neurons affects neuronal excitability and results from metabolic H^+^ generation during repetitive firing [122]. Neuronal ZnR/GPR39 activation following release of Zn^2+^, concomitant with the neurotransmitter, resulted in increased NHE activity, thus relieving the metabolic acidification [109]. However, acidification of neuronal surfaces, by upregulating NHE activity, may contribute to tissue acidosis during ischemic neuronal injury [123]. Interestingly, ZnR/GPR39 itself is inactive at acidic pH [109], suggesting a homeostatic mechanism that can attenuate ZnR/GPR39 activation of NHE and excessive tissue acidification. 

The K^+^/Cl^−^ cotransporters (KCC) family is responsible for mediating Cl^−^ efflux and thereby maintaining cell volume, as well as transepithelial ion transport and neuronal excitability [124]. These transporters are highly regulated via their phosphorylation and changes in surface expression [125,126]. In neurons, KCC2 is crucial for mediating Cl^−^ efflux and thereby rendering the GABA_A_ and glycine receptors inhibitory [127,128,129,130]. Activation of ZnR/GPR39 results in enhanced K^+^-dependent Cl^−^ transport, which is mediated by KCC2 [62,131]. This Zn^2+^-dependent upregulation is abolished in the absence of ZnR/GPR39, or its downstream Ca^2+^ and MAPK activation. Moreover, Gαq-dependent signaling triggered by ZnR/GPR39 enhances KCC2 surface expression and thereby upregulates KCC2-dependent Cl^−^ transport [62]. Similar upregulation of K^+^-dependent Cl^−^ transport was also monitored following ZnR/GPR39 activation in colonocytes [91]. Loss of Cl^−^ and Na^+^ into the colon lumen, via CFTR (cystic fibrosis transmembrane conductance regulator) upregulation for example, produces the driving force for water loss, thereby inducing diarrhea [132]. Yet, Cl^−^ absorption pathways are not fully identified. Activation of ZnR/GPR39 in native colon epithelial tissue or in colonocytic cell lines resulted in activation of KCC1, which was mitogen activated kinase (MAPK)-dependent [91]. Moreover, KCC1 expression was shown to be basolateral, thereby providing a pathway for modulation of Cl^−^ absorption in the colon.

### 4.2. ZnR/GPR39 Regulates Tight Junction Formation

Formation of epithelial barriers strongly depends on expression of junctional proteins, such as E-cadherin of the adherens junctions and zonula occludens-1 (ZO-1) or occludin of the tight junctions. This physical barrier is essential for the function of all epithelia and is particularly important in regions exposed to pathogens, such as the digestive tract. A role for Zn^2+^ in modulating colon epithelial tight junctions was previously described, but the prolonged application in that study may have resulted in changes of intracellular Zn^2+^ and not only activation of ZnR/GPR39 [133,134]. Using siRNA silencing of ZnR/GPR39 in Caco-2 colonocytic cell line revealed that ZnR/GPR39 was essential for Zn^2+^-dependent upregulation of tight junction formation, thus establishing that ZnR/GPR39 has a specific role in enhancing tight junctional complexes and epithelial barrier function [89]. It was further established that these colonocytic cells release Zn^2+^ in a manner that activates the ZnR/GPR39-dependent formation of the barrier, since a chelator of extracellular Zn^2+^ attenuated tight junction formation. Colon from ZnR/GPR39 deficient mice exhibited diminished expression level for the tight junction protein occludin, further revealing an important role of ZnR/GPR39 in barrier formation in vivo [90]. This loss of tight junctions may underlie some of the immune system effects associated with Zn^2+^ deficiency: as the permeation of pathogens is easier, inflammation may be prevalent during Zn^2+^ deficiency. A recent study showed that Zn^2+^ enhanced the expression of protein kinase C ζ (PKCζ), which was associated with ZnR/GPR39 levels, and linked to tight junction formation during *Salmonella enterica serovar Typhimurium* infection [135].

## 5. A Role for ZnR/GPR39 in Disease

### 5.1. ZnR/GPR39 in Wound Healing

Perhaps the oldest known use of zinc as a treatment is in dermal ointments for enhancing wound healing [104,105,106,107]. Zinc has been associated with proliferating tissues and is indeed accumulated in the skin [136]. Zinc transporters, i.e., ZIP4 (Zrt-Irt-like protein) or ZIP7, knockdown or mutations in these proteins also reveal an important role for these Zn^2+^ homeostatic proteins in skin formation during development [137]. Activation of ZnR/GPR39 in primary keratinocytes and in HaCaT cells suggested that Zn^2+^ may trigger this receptor signaling and may be the missing link between topical application of zinc and wound healing [41,52]. Indeed, the pro-proliferation/migration pathways were activated by ZnR/GPR39: ERK1/2 phosphorylation was increased via ZnR/GPR39-dependent activation of PKC and PI3K. In a scratch assay model, silencing of ZnR/GPR39 expression or activity inhibited the Zn^2+^-dependent increased rate of scratch closure [41]. One of the suggested benefits of zinc application was associated with anti-inflammatory effects. The ZnR/GPR39-dependent activation of NHE in keratinocytes induces acidification of the extracellular region. Such acidification is essential for reducing barrier permeability in the skin [120]. Hence NHE activation and the subsequent acidification by the ZnR/GPR39 may also exert an anti-inflammatory effect. Finally, if paracrine release of ATP following ZnR/GPR39 activation [58] also occurs in keratinocytes, it suggests another mechanism to increase the proliferation of neighboring fibroblasts that do not express ZnR/GPR39. Activation of cellular signaling by ZnR/GPR39 may affect numerous pathways and Zn^2+^ binding proteins. As such, a role for MG53, a Zn^2+^ binding protein, has been associated with myoblasts’ cell membrane recovery following permeation of Zn^2+^ into the cells [138]. The ZnR/GPR39 has also been described in myogenic processes, but the role of Zn^2+^ in this aspect has not been addressed [74], hence future studies on the role of ZnR/GPR39 in muscle cell recovery would be of interest. 

### 5.2. Diarrhea and Inflammatory Bowel Diseases

Prominent roles of Zn^2+^ include attenuation of diarrhea and amelioration of symptoms of inflammatory ulcerative disease, such as Crohn’s disease and colitis [139,140,141,142,143]. Initial breakdown of tight junctions is considered a trigger to recurrence of inflammatory bowel diseases. The ZnR/GPR39-dependent enhancement of junctional complex proteins ZO-1 and occludin [69,89] suggested that ZnR/GPR39 may be involved in ameliorating symptoms of inflammatory bowel diseases. Indeed, ZnR/GPR39 deficient mice showed increased susceptibility to dextran sodium sulfate (DSS) model of colitis [90]. Even more profound was the effect of ZnR/GPR39 during a recovery phase. ZnR/GPR39 expression was essential for rapid recovery of the epithelial layer, via increased proliferation and crypt formation, and formation of the physical barrier, via increased expression of occludin. The benefit of Zn^2+^ treatment in inflammatory bowel disease is controversial. The results described here suggest that during bouts of the inflammatory state the epithelial erosion and loss of ZnR/GPR39 on the epithelial barrier may render Zn^2+^ inefficient, yet if provided during remission Zn^2+^, via ZnR/GPR39, may extend this period. In fact, ZnR/GPR39 expression in the epithelial cells may serve as a therapeutic target that can be specifically activated to extend the remission periods.

Maintenance of osmotic gradients, for proper water movement, is mediated by ion transporters found on the epithelial cells [144]. In diarrhea, impaired transporters function results in excessive loss of Na^+^ and Cl^−^ into the lumen and subsequent water loss. The Zn^2+^ and ZnR/GPR39 upregulation of Na^+^/H^+^ exchanger activity [57,69] can serve to enhance uptake of Na^+^ from the lumen. Indeed many previous studies showed that the colonocytic apical NHE3 upregulation enhances Na^+^ absorption and thereby reduces water loss and diarrhea [144,145,146]. In addition, activation of a basolateral KCC1 by ZnR/GPR39 increases absorption of Cl^−^, which is also essential to reducing fluid loss. Cholera toxin infection, a common cause of diarrhea, induced significantly worse diarrhea in *GPR39* knockout mice, lacking ZnR/GPR39 signaling, compared to WT mice [91]. Thus, ZnR/GPR39 activation can reduce fluid loss during the disease, but reduced luminal Zn^2+^, which may be a dietary or disease-mediated condition, may diminish the protective effect of this pathway. While Zn^2+^ is suggested by the World Health Organization (WHO) as an important supplement to treat diarrhea [142,147], ZnR/GPR39 is a novel and specific target that may be more effectively targeted. 

### 5.3. Epilepsy

Several studies linked the loss of synaptic Zn^2+^ or Zn^2+^ deficiency with increased incidence of seizures [148,149,150,151]. Despite a well-known role for Zn^2+^ in modulating numerous excitatory and inhibitory post synaptic targets, how synaptically released Zn^2+^ can affect epileptogenesis was not clear. Nevertheless, the major phenotype of the *ZnT3* knockout mice, lacking synaptic Zn^2+^, is enhanced sensitivity to kainate-induced or febrile hyperthermia induced seizures [152,153]. This indicated that synaptic Zn^2+^ itself does have a role in epilepsy. The results showing regulation of Cl^−^ transport by ZnR/GPR39 activation of KCC2, taken together with the prominent role of loss of KCC2 function in increasing seizure susceptibility [128,154,155], suggested that ZnR/GPR39 may play a role in epilepsy via this pathway. Indeed, *GPR39* knockout animals, lacking ZnR/GPR39 signaling, exhibit enhanced susceptibility to kainate-induced seizures, with significantly higher behavioral seizure severity scores and more seizures over longer periods of time compared to wild-type controls [156]. Kainate-induced upregulation of KCC2 activity is dependent on Zn^2+^, which is released by the increased firing under these enhanced excitability conditions. Moreover, ZnR/GPR39 signaling via the Gαq and subsequent MAPK pathway are required for increased KCC2 activity and thereby inhibitory tone. Thus the homeostatic role of ZnR/GPR39, activated by Zn^2+^ co-released with glutamate, is essential during excessive firing to reduce excitatory activity via enhancing GABAergic responses. In contrast, loss of this signaling in the absence of synaptic Zn^2+^ or ZnR/GPR39 may result in epileptogenesis [53]. A similar effect on increasing inhibitory neuronal signaling is monitored in the dorsal cochlear neurons, where ZnR/GRP39 activation enhances endocannabinoid release and reduces excitatory glutamate release [63]. In addition, enhanced excitability and thereby seizure activity has been associated with neuronal acidification and loss of Na^+^/H^+^ exchanger (NHE) activity [157,158]. Thus ZnR/GPR39-dependent upregulation of NHE activity, which was monitored in primary neurons, may also link the receptor to reduced seizures [109]. In Alzheimer’s disease, Aβ oligomers interact with Zn^2+^ [159,160], thus lowering levels of labile Zn^2+^. Indeed, in the presence of Aβ, the ZnR/GPR39-dependent Ca^2+^ responses in primary neurons were significantly reduced and resulted in much lower MAPK activation [161]. This decrease in ZnR/GPR39-dependent signaling, reducing the homeostatic activation of KCC2, may serve as a link to the increased incidence of seizure found in Alzheimer’s disease patients compared to the general population. 

### 5.4. Depression

Zinc deficiency is associated with neurological and psychiatric disorders [162]; however, it is not yet clear if the decrease in Zn^2+^ results from aberrant intake, especially in depression, when appetite is lost and general uptake of nutrients is low, or is a cause of the disorder. Several studies reported a role for ZnR/GPR39 in depression, based on apparent changes in the expression level of this receptor following Zn^2+^-deficiency that were correlated with behavioral changes, also in suicide victims [163,164]. Changes in ZnR/GPR39 expression were also shown following treatment with monoaminergic inhibitors, such as used to treat depression, thus suggesting a link between the receptor and this disease. Surprisingly, despite the extensive use of antibodies against ZnR/GPR39 in this study, the antibodies were not verified in *GPR39* knockout mice [165]. A role for ZnR/GPR39 in the regulation of the CREB/BDNF/TrkB (cyclic AMP response element binding protein/brain-derived neurotrophic factor/tyrosine receptor kinase B) pathway, and thereby in depression, has also been postulated, though it is not clear at present how Gαq signaling activates this pathway or whether these effects are lost in ZnR/GPR39 deficient mice [166,167].

### 5.5. Insulin Secretion

Pancreatic β-cells contain vesicular Zn^2+^ that is released together with insulin [168]. Several studies have highlighted a role for Zn^2+^ in the regulation of β-cell function and glucagon release [169,170,171]. The Zn^2+^ transporter ZnT8 is responsible for transporting Zn^2+^ into the insulin vesicles, and a mutation in this transporter of an Arg replacing Trp325 is associated with increased risk of developing type 2 diabetes [172]. Thus a role for ZnR/GPR39 in this tissue may have important physiological implications in the regulation of the Zn^2+^ releasing β-cells or neighboring cells within the islets of Langerhans. Knockout of ZnR/GPR39 does not immediately produce a phenotype under baseline conditions, and the knockout mice show normal insulin secretion. However, when fed a sucrose-rich diet, older mice show increased glucose levels and decreased insulin compared to the wild type [173]. Similarly, higher glucose levels were monitored in *GPR39* knockout mice fed a high-fat diet [174]. In agreement, overexpression of ZnR/GPR39 in β-cells resulted in protection from streptozotocin-induced diabetes [175]. A recent study showed ZnR/GPR39 expression and Zn^2+^-dependent Ca^2+^ release in association with Zn^2+^-dependent insulin secretion [176]. Yet how ZnR/GPR39 activity regulates insulin secretion and whether this is an autocrine effect of endogenous Zn^2+^ released from the β-cells is still poorly understood. 

### 5.6. Defects in Bone Composition

Zinc is accumulated in bone and plays a role in the dynamic maintenance of the structure of bones. Supplementation with dietary zinc enhances the strength of bones, but an underlying mechanism is not available. While several zinc transporters of the ZIP family have been associated with skeletal function [137], a role for ZnR/GPR39 was not described. Using *GPR39* knockout mice, a recent study indicates that this receptor is important for osteoblast differentiation [61]. Hence, mice lacking ZnR/GPR39 showed impaired bone composition with decreased collagen content, likely involving ADAMTS metalloproteinase, which regulates collagen processing [61]. Most importantly, ZnR/GPR39 deficient osteoblasts showed lower *ZIP13* expression, linking ZnR/GPR39 and Zn^2+^ transporters for the first time. Future studies aiming to determine how ZnR/GPR39 modulates Zn^2+^ transporters’ activity or expression can provide a more complete picture of the network of zinc homeostasis and its physiological implications. 

### 5.7. ZnR/GPR39 in Cancer

Increased cell proliferation and migration, as well as the activation of MAPK and AKT, suggest a possible role for ZnR/GPR39 in cancer. Activation of ZnR/GPR39 signaling was monitored in androgen-independent, but not androgen-dependent, prostate cancer cells [59]. Extracellular Zn^2+^ via activation of ZnR/GPR39 in the prostate cancer cell line PC-3 enhances expression of S100A4 [84], a protein that is thought to enhance metastatic prostate cell proliferation and angiogenesis [177]. Other studies that associated ZnR/GPR39 expression in epithelial cells with cancer did not employ changes in extracellular or dietary Zn^2+^ to specifically study whether signaling pathway activation or Zn^2+^-dependent processes are affected in the cancer cells. These studies nevertheless indicate the importance of this receptor as a therapeutic target for cancer treatment. As such, GPR39 was overexpressed in primary human esophageal squamous cell carcinomas and its silencing reduced the tumorigenicity of these cells [178]. A recent study suggested that GPR39 expression is modulated in gastric adenocarcinoma [179], yet this study applied a previously incorrectly suggested ligand of GPR39 and not Zn^2+^ [180]. Interestingly, a link between the ZnR/GPR39 and mRNA levels of the Zn^2+^ transporter *ZIP13* was recently shown in bone [61], but whether ZnR/GPR39 regulates other members of the ZIP family of Zn^2+^ transporters is unknown. Such a link between ZnR/GPR39 and ZIP transporters may further link the receptor to tumorigenesis. For example, ZIP6 and ZIP7 overexpression in breast cancer has been previously shown [181,182], and ZIP4 has recently been associated with ovarian stem cell growth and carcinoma [183]. Future studies aiming to specifically test the role of ZnR/GPR39 in cancer tissue and the link to ZIP transporters expression are of major interest and can provide a novel target for therapeutic tools. 

## 6. Conclusions

ZnR/GPR39 is an important regulator of Zn^2+^-dependent signaling, functional in numerous epithelial cells, bone cells, and neurons—all tissues associated with Zn^2+^ homeostasis. Transient changes in extracellular Zn^2+^ occur during physiological activity and are sufficient to activate ZnR/GPR39. While dietary or serum zinc itself has been suggested to affect the physiological function or pathological conditions in these tissues, these changes in zinc concentration do not directly reflect local or cellular changes in the concentrations of the ionic Zn^2+^. In addition, Zn^2+^ interacts with a multitude of intracellular or extracellular proteins and modulates their activity, as described in the introduction; therefore, changes in Zn^2+^ concentration may affect many proteins and cellular functions and not just ZnR/GPR39 activity. Thus, this micronutrient is a poor therapeutic compound with inconsistent effects. However, elucidation of ZnR/GPR39 as a regulator of Zn^2+^-dependent cellular signaling can offer a novel handle to effective therapeutic approaches that will depend on ZnR/GPR39 agonists. Of note, ZnR/GPR39 is a member of the G-protein coupled receptor family, which is currently considered a major candidate for targeted therapies [184,185]. Finally, what regulates the activity of Zn^2+^ transporters is only partially understood; for example, it was previously shown that intracellular Zn^2+^ activation of metal-responsive elements regulates ZnT expression or that phosphorylation of ZIP regulates their expression [24,25]. In this context, a possible link between ZnR/GPR39 and the transporters may be a key to understanding Zn^2+^ homeostasis and is an important aim for future studies. Thus ZnR/GPR39 may serve as a specific and efficacious handle to modulate Zn^2+^ homeostatic proteins and signaling, thereby ameliorating physiological processes to enhance recovery.
